# Age-related differences in the respiratory microbiota of chickens

**DOI:** 10.1371/journal.pone.0188455

**Published:** 2017-11-22

**Authors:** Laura Glendinning, Gerry McLachlan, Lonneke Vervelde

**Affiliations:** 1 Developmental Biology, The Roslin Institute and Royal (Dick) School of Veterinary Studies, University of Edinburgh, Edinburgh, Midlothian, United Kingdom; 2 Infection and Immunity, The Roslin Institute and Royal (Dick) School of Veterinary Studies, University of Edinburgh, Edinburgh, Midlothian, United Kingdom; Argonne National Laboratory, UNITED STATES

## Abstract

In this era of next generation sequencing technologies it is now possible to characterise the chicken respiratory microbiota without the biases inherent to traditional culturing techniques. However, little research has been performed in this area. In this study we characterise and compare buccal, nasal and lung microbiota samples from chickens in three different age groups using 16S rRNA gene analysis. Buccal and nasal swabs were taken from birds aged 2 days (n = 5), 3 weeks (n = 5) and 30 months (n = 6). Bronchoalveolar lavage (BAL) samples were also collected alongside reagent only controls. DNA was extracted from these samples and the V2-V3 region of the 16S rRNA gene was amplified and sequenced. Quality control and OTU clustering were performed in mothur. Bacterial DNA was quantified using qPCR, amplifying the V3 region of the 16S rRNA gene. We found significant differences between the quantity and types of bacteria sampled at the three different respiratory sites. We also found significant differences in the composition, richness and diversity of the bacterial communities in buccal, nasal and BAL fluid samples between age groups. We identified several bacteria which had previously been isolated from the chicken respiratory tract in culture based studies, including lactobacilli and staphylococci. However, we also identified bacteria which have not previously been cultured from the respiratory tract of the healthy chicken. We conclude that our study can be used as a baseline that future chicken respiratory microbiota studies can build upon.

## Introduction

Many studies have been performed which have used 16S rRNA gene analysis to study the human respiratory microbiota and it has been recognised that these communities of bacteria are highly important in the maintenance of respiratory health [1].

However, to our knowledge only one study has been published which has studied the respiratory microbiota of the healthy chicken using 16S rRNA gene analysis [2]. In contrast, the importance of the gut microbiota with regard to growth performance and reduction of pathogen load of poultry is well recognised and the composition and dynamics of the microbial communities in the gastrointestinal tract have been studied in more detail using next generation sequencing [3–6]. In mammals, the composition of the respiratory microbiota is associated with disease severity [7] and future risk of developing respiratory disease [8]. Vaccination against respiratory pathogens such as *Streptococcus pneumonia* has also been linked to changes in the respiratory bacterial flora as the suppression of vaccine targets within these communities can lead to the proliferation of other bacterial species [9,10]. Members of the respiratory microbiota also contribute to the maintenance of the mucosal immune system [11], and alterations in the local immune environment, such as increased inflammation due to acute lung injury, can also lead to changes in the bacterial communities present and the outgrowth of opportunistic pathogens [12].

Investment into understanding the composition of bacterial communities, their effect on the immune status of the respiratory tract and the interaction with pathogens in chickens would likely have a positive impact on poultry health, given the fact that most viruses in chicken enter through the respiratory tract and the live attenuated vaccines to prevent them are given by spray or oculo/nasal route.

Using culture based methods, a wide variety of bacteria and fungi have been isolated from the respiratory tracts of healthy chickens [13–15]. However, these studies are only able to isolate and characterise those microbes which can be cultured and identified under laboratory conditions. By sequencing the bacterial 16S rRNA genes present in a sample it is possible to identify the bacteria it originally contained without the need for culturing and at a far smaller cost than using shotgun metagenomics.

This is the first study to compare buccal, nasal and lung microbiota samples from chickens of different ages using 16S rRNA gene analysis.

## Materials and methods

### Study design

Novogen Brown chickens were bred and housed at the National Avian Research Facility in Edinburgh (UK). The chickens were housed in groups in floor pens with wood shavings bedding and received food and water ad libitum. The birds in this study were 2 days (n = 5), 3 weeks (n = 5) and 30 months (n = 6) of age and all birds were considered healthy by physical examination. The 30-month-old birds were vaccinated according to S1 Table, the younger birds were not vaccinated. Chicken husbandry conditions are described in S2 Table. Animals were housed in premises licensed under a UK Home Office Establishment License within the terms of the UK Home Office Animals (Scientific Procedures) Act 1986. Housing and husbandry complied with the Code of Practice for Housing and Care of Animals Bred, Supplied or Used for Scientific Purposes and were overseen by the Roslin Institute Animal Welfare and Ethical Review Board. Animals were culled by schedule one methods authorized by the Animals (Scientific Procedures) Act 1986. Birds were euthanized by cervical dislocation.

To minimise contamination from bacterial DNA (from dead bacteria) originating from the lab environment, all of the reagents and equipment used during sampling were first treated with UV. All procedures were carried out in a lamina flow cabinet which had been treated with DNA Zap solution (DNAZap PCR DNA Degradation Solutions, Thermo Fisher Scientific). All sampling equipment was also treated with DNA Zap.

Buccal and nasal swabs were taken using plastic feeding tubes (20 ga x 38 mm, sterile, Instech). Prior to sampling of bronchoalveolar (BAL) fluid, for each chicken a negative control containing only 2.5 ml sterile phosphate buffered saline (PBS) was produced by passing the PBS through the same needle, syringe and tubing which was then used to collect BAL from the chicken. These controls underwent DNA extraction and PCR amplification alongside BAL samples in order to identify contaminating bacterial DNA. BAL sampling was performed by exposing the trachea and making a small incision. While chickens do not have alveoli, we will use the phrase BAL fluid to refer to these samples as this is the most commonly used terminology in the literature. A 20G, 0.9 x 52 mm needle was sheathed in plastic tubing and inserted into the trachea. PBS was passed through the needle and into the lungs before being withdrawn. 1.5 ml of PBS was used for sampling the 2 day old birds, 2.5 ml for the 3 week old birds and 10 ml for the 30 month old birds. For two birds (3 week old 4828 and 30 month old 4329) BAL samples were not able to be collected due to burst air sacs. Not including these birds, on average 1.12 ml of BAL fluid was collected from the 2 day old birds, 0.88 ml from the 3 week old birds and 5.6 ml from the 30 month old birds. Buccal and nasal swabs, PBS controls and BAL fluids were immediately frozen on dry ice then stored at -80°C until DNA extraction.

### DNA extraction and 16S rRNA gene amplification

Samples were randomised into three DNA extraction batches. DNA extraction was performed as described previously [16] using the PowerSoil® DNA Isolation Kit (Mo Bio). DNA extraction reagent only controls were included for each batch of DNA extractions (two controls were included in the third extraction batch, referred to as extraction controls 3 and 4). The V2-V3 region of the 16S rRNA gene was amplified via PCR as described previously [16]. Briefly, a nested PCR protocol was performed using the V1-V4 primers 28F (‘5–175 GAGTTTGATCNTGGCTCAG-3’) and 805R (‘5-GACTACCAGGGTATCTAATC-3’) followed by the V2-V3 primers 104F (‘5-GGCGVACGGGTGAGTAA-3’) and 519R (‘5–177 GTNTTACNGCGGCKGCTG-3’) with Illumina adaptor sequences and barcodes.

### Sequencing and data analysis

Amplicons were sequenced using an Illumina Miseq producing paired-end 250 base-pair reads. Primers were removed using cutadapt. Quality control and operational taxonomic unit (OTU) clustering were performed within mothur [17] following a protocol developed by the mothur creators [18] as previously described [16]. OTUs were clustered using a database-dependent approach and were then subsampled (S3 Table). Good’s coverage was used to estimate sample coverage [19]. Analysis of molecular variance (AMOVA) was used to determine if there were significant differences in the bacterial communities between sample groups. AMOVA is a non-parametric test which tests if groups of samples cluster significantly separately by their bacterial community compositions [20]. The Kruskal-Wallis test was used to detect significant differences in richness (Chao 1 index) and diversity (Inverse Simpsons diversity index) between bacterial communities. The Chao 1 index is based upon the amount of rare OTUs which are present in a sample; a high Chao 1 richness index value indicates a high number of rare OTUs. The Inverse Simpsons diversity index takes into account both OTU abundance and the number of OTUs present in a sample; an increase in the Inverse Simpsons Index indicates an increase in species richness and evenness, and thereby in diversity. Indicator analysis was used to identify OTUs which were significantly more abundant in specific sample groups [21]. Heatmaps were generated in R Version 3.2.2 (R Foundation for 212 Statistical Computing), a boxplot for qPCR data was constructed in SPSS Statistics 21 (IBM Analytics) and a stacked bar chart was created in Excel 2013 (Microsoft). Principle coordinate analysis graphs (PCOA) were constructed within mothur to visualise sample clustering by bacterial community composition.

The unassembled sequencing reads used to generate the data in this paper can be found at Bioproject accession number PRJNA393945.

### qPCR

Quantification of the V3 region of the 16S rRNA gene was performed as described previously [22] using the primers UniF340 (‘5–222 ACTCCTACGGGAGGCAGCAGT-3’) and UniR514 (‘5-ATTACCGCGGCTGCTGGC-3’) at a final concentration of 0.4 μM using the LightCycler 480 SYBR green I master mix (Roche Applied Science).

## Results

### Quality control

The V2-V3 region of the 16S gene was sequenced and quality control was performed on these sequences. 47% of sequences were removed during quality control. Per sample the average number of reads after quality control was 100,531 ± 40,849 (mean ± standard deviation (SD)). The lowest Good’s coverage value for any sample was 0.998. This means that for all samples at least 99.8% of the bacteria which were in the original samples were identified. The sequence error rate was 0.25% and a total of 812 bacterial OTUs were identified.

The six most abundant bacterial OTUs on average in DNA extraction reagent only controls were Nostocophycideae (18.9%), Scytonemataceae (13.1%), *Actinobacillus* (12.7%), *Anaerococcus* (8.3%), *Enhydrobacter* (4.2%) and *Pelomonas* (3.8%). The most abundant bacterial OTUs on average in PBS controls were *Pseudomonas* (29.7%), Nostocophycideae (17.4%), Scytonemataceae (9.5%), *Lactobacillus* (2.9%), *Erwinia* (2.5%) and *Methylobacterium* (2.5%).

### Comparing buccal, nasal and lung samples

**Fig 1:**
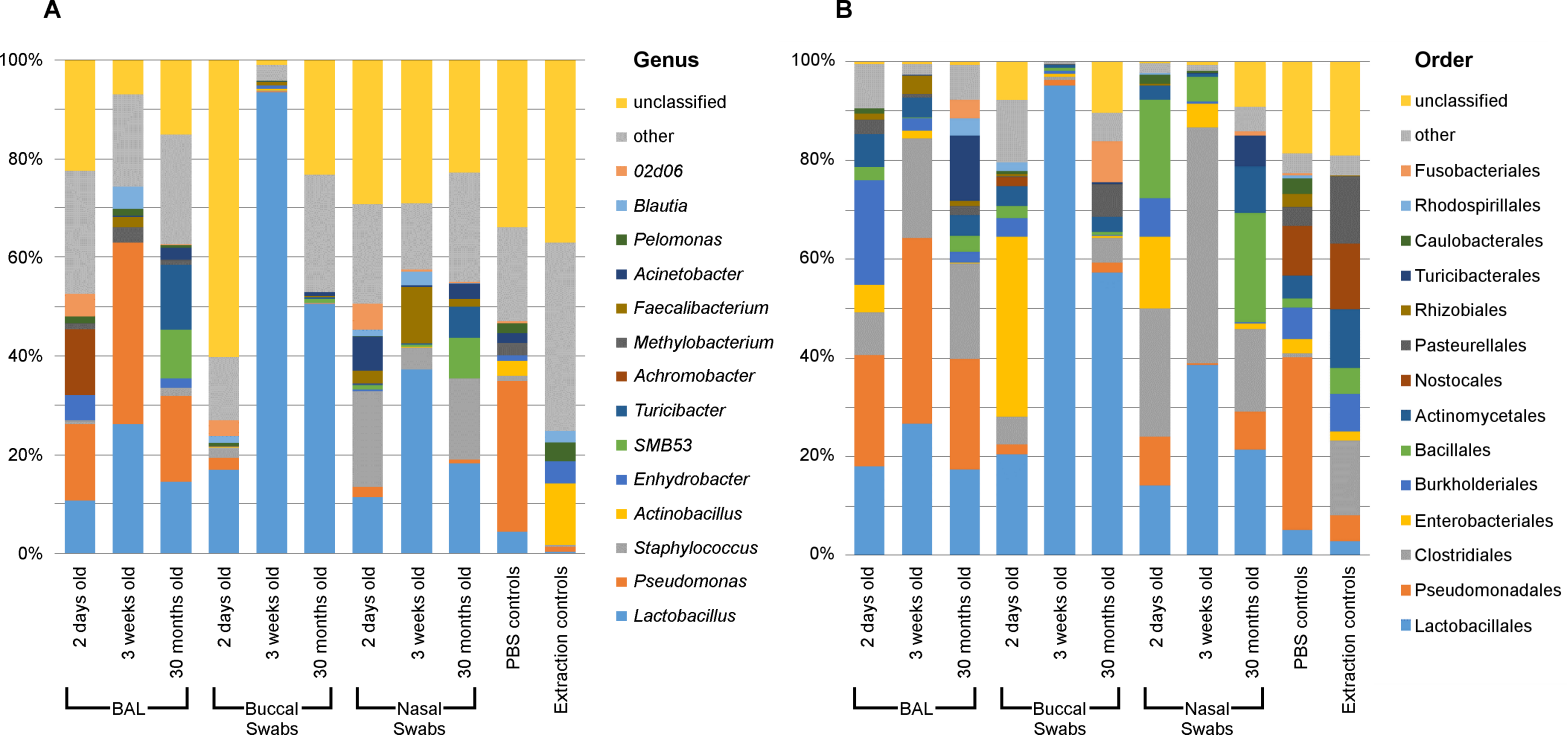
Bacterial genera in respiratory samples from chickens. The most abundant bacterial genera (A) and orders (B) on average in respiratory samples from chickens of different ages and in PBS and DNA extraction reagent controls.

Buccal, nasal and BAL samples clustered significantly separately by their bacterial community compositions (AMOVA: P<0.001) (Fig 1). Both richness and diversity were also significantly different between sample types (Kruskal-Wallis Test: P<0.001) (Table 1).

**Table 1:** Richness and diversity of bacterial communities in different sample types.

Sample type	Chao (richness) (median ± SD)	Inverse Simpsons (diversity) (median ± SD)
BAL	All: 86.74 ± 41.6	All: 5.66 ± 3.59
	2 days old: 80.27 ± 19.68	2 days old: 5.52 ± 3.92
	3 weeks old: 76.23 ± 11.82	3 weeks old: 2.83 ± 2.96
	30 months old: 97.97 ± 63	30 months old: 7.85 ± 3.44
Buccal swabs	All: 88.63 ± 42.1	All: 4.24 ± 7.03
	2 days old: 73.84 ± 50	2 days old: 2.65 ± 12.71
	3 weeks old: 76.06 ± 17	3 weeks old: 3.63 ± 0.88
	30 months old: 126.81 ± 28	30 months old: 5.93 ± 1.92
Nasal swabs	All: 162.45 ± 73.7	All: 9.33 ± 4.46
	2 days old: 117.1 ± 46.5	2 days old: 6.25 ± 3.21
	3 weeks old: 150.2 ± 19.59	3 weeks old: 8.55 ± 4.20
	30 months old: 267 ± 59.3	30 months old: 10.84 ± 5.68
PBS and DNA extraction reagent controls	All: 80.90 ± 13.72	All: 3.68 ± 2.98

The V3 region of the 16S rRNA gene was quantified in our samples using qPCR in order to ascertain how much bacterial DNA was in these samples. Different sample types contained significantly different quantities of bacterial DNA (Kruskal-Wallis: P<0.001). On average, samples contained the following concentrations of this bacterial amplicon (mean ± SD): buccal swabs (6.02x10^-3^ ± 1.42x10^-2^ ng/μl), nasal swabs (2.59x10^-3^ ± 6.25x10^-3^ ng/μl), BAL fluids (2.37x10^-2^ ± 8.06x10^-2^ ng/μl), PBS controls (1.3x10^-4^ ± 1.5x10^-5^ ng/μl), DNA extraction reagent controls (1.32x10^-4^ ± 1.5x10^-5^ ng/μl) and water controls (1.31x10^-4^ ± 1.0x10^-5^ ng/μl).

### Buccal microbiota

**Fig 2:**
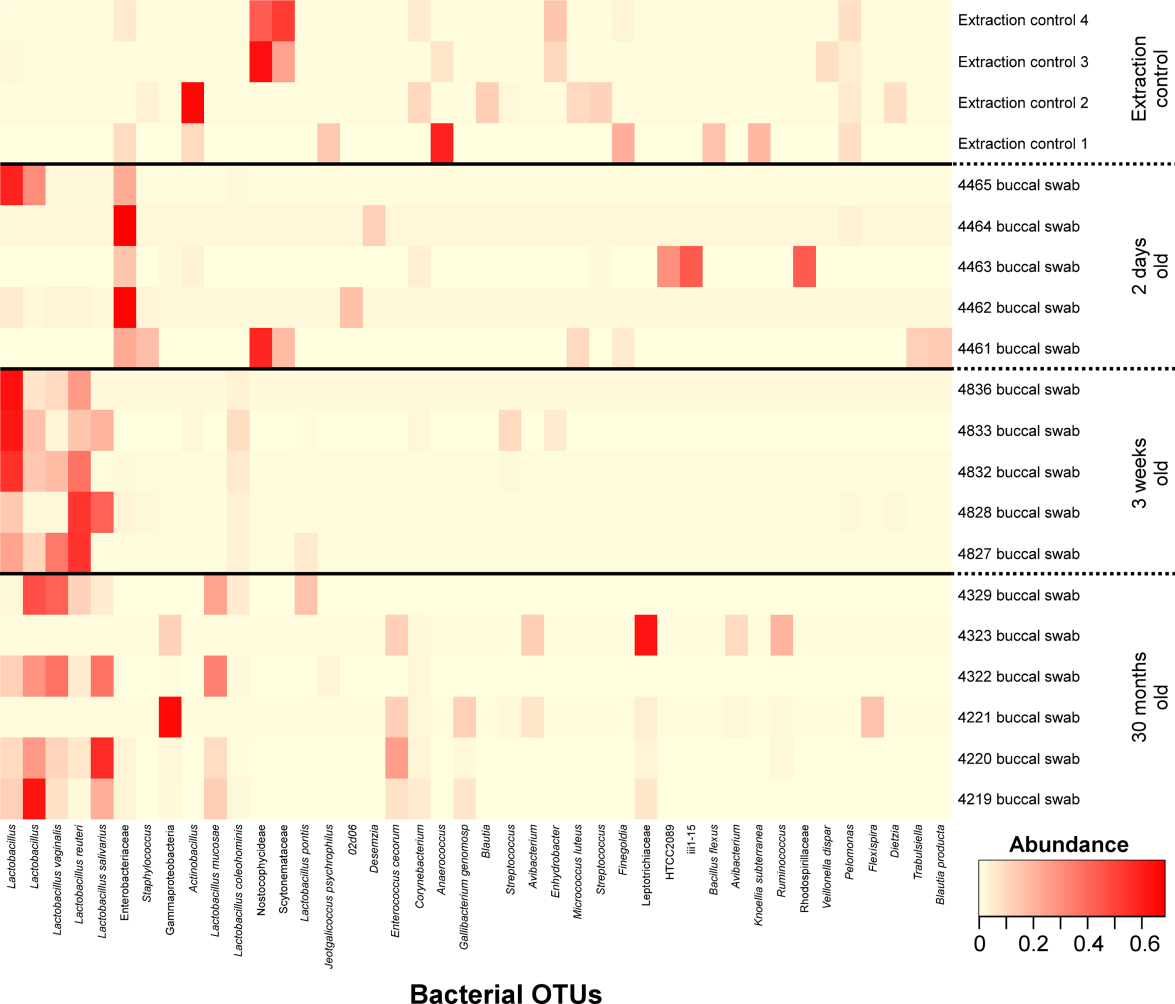
Heatmap of buccal swab samples grouped by animal age. Bacterial OTUs were included where they had an abundance of ≥5% in at least one sample. DNA extraction kit reagent controls are labelled as Extraction control *n*.

**Fig 3:**
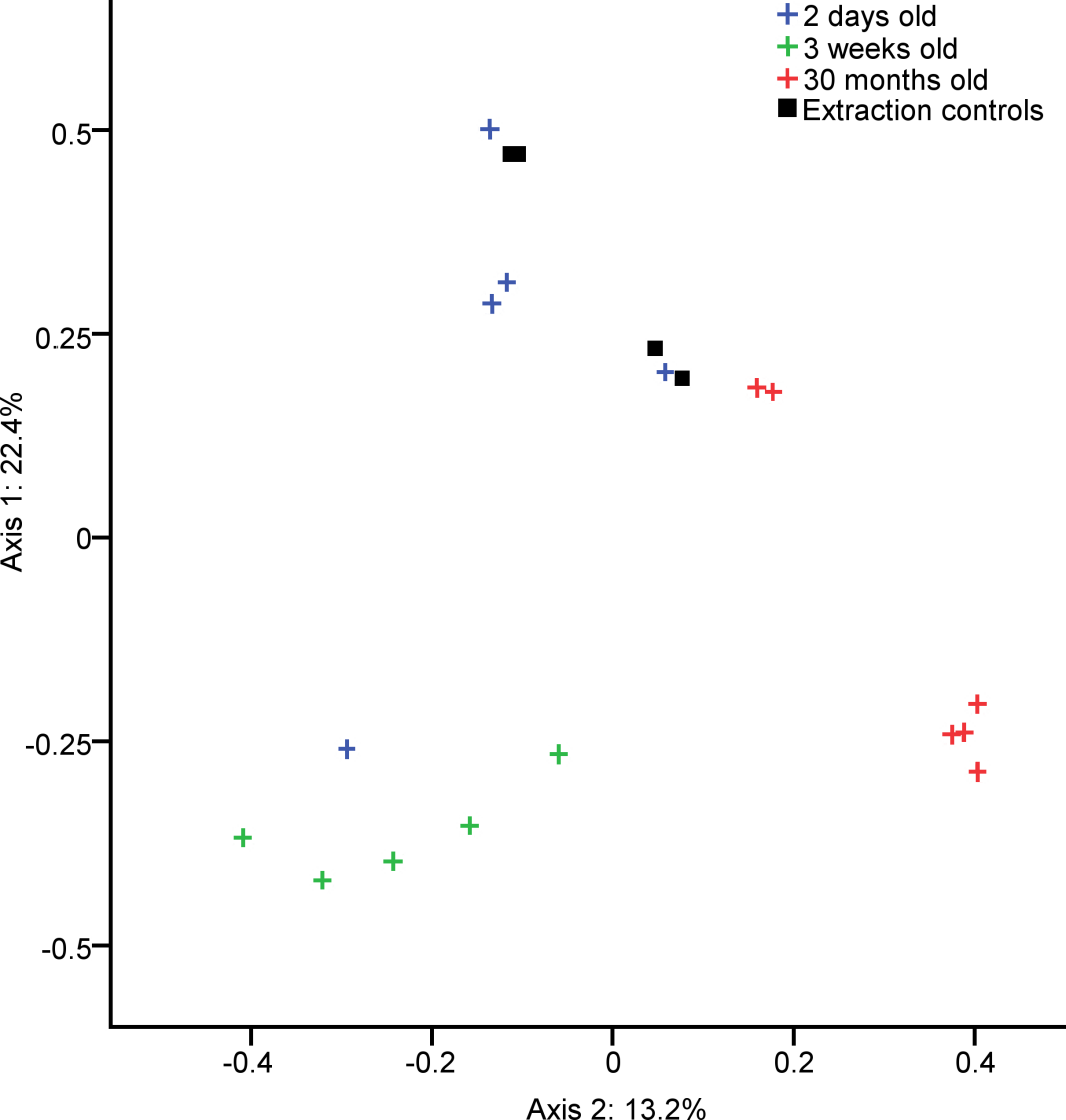
Clustering of chicken buccal swab samples according to age. PCOA graph showing the significantly separate clustering by community composition of the bacterial communities in buccal swabs from chickens of different ages (AMOVA: P<0.001).Each axis label shows the total percentage of variability between samples which is represented by that axis.

The most common bacterial OTUs found on average in buccal swabs were *Lactobacillus* (15.3%), Enterobacteriaceae (10.9%), *Lactobacillus reuteri* (10.0%), *Lactobacillus* (9.6%), *Lactobacillus vaginalis* (6.7%) and *Lactobacillus salivarius* (6.7%).) (Fig 2). Samples clustered by their bacterial community compositions according to age group (AMOVA: P<0.001) (Fig 3). The richness of these bacterial communities was also significantly different between age groups (Kruskal-Wallis Test: P = 0.02), with richness rising with age, but age groups did not differ significantly in diversity. Bacterial OTUs which are indicative of specific age groups can be found in S4 Table.

### Nasal microbiota

**Fig 4:**
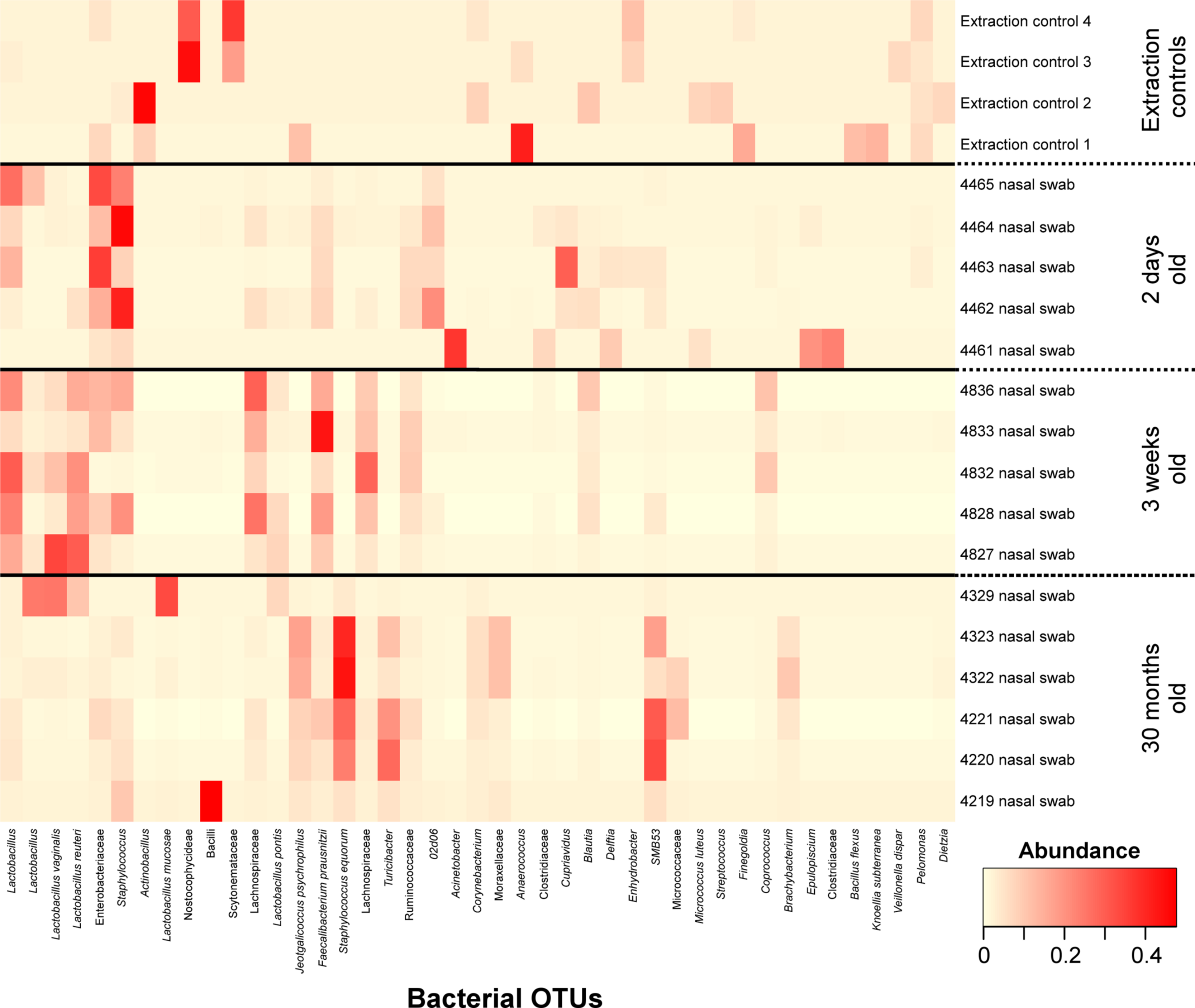
Heatmap of nasal swab samples grouped by animal age. Bacterial OTUs were included where they had an abundance of ≥5% in at least one sample. DNA extraction kit reagent controls are labelled as Extraction control *n*.

**Fig 5:**
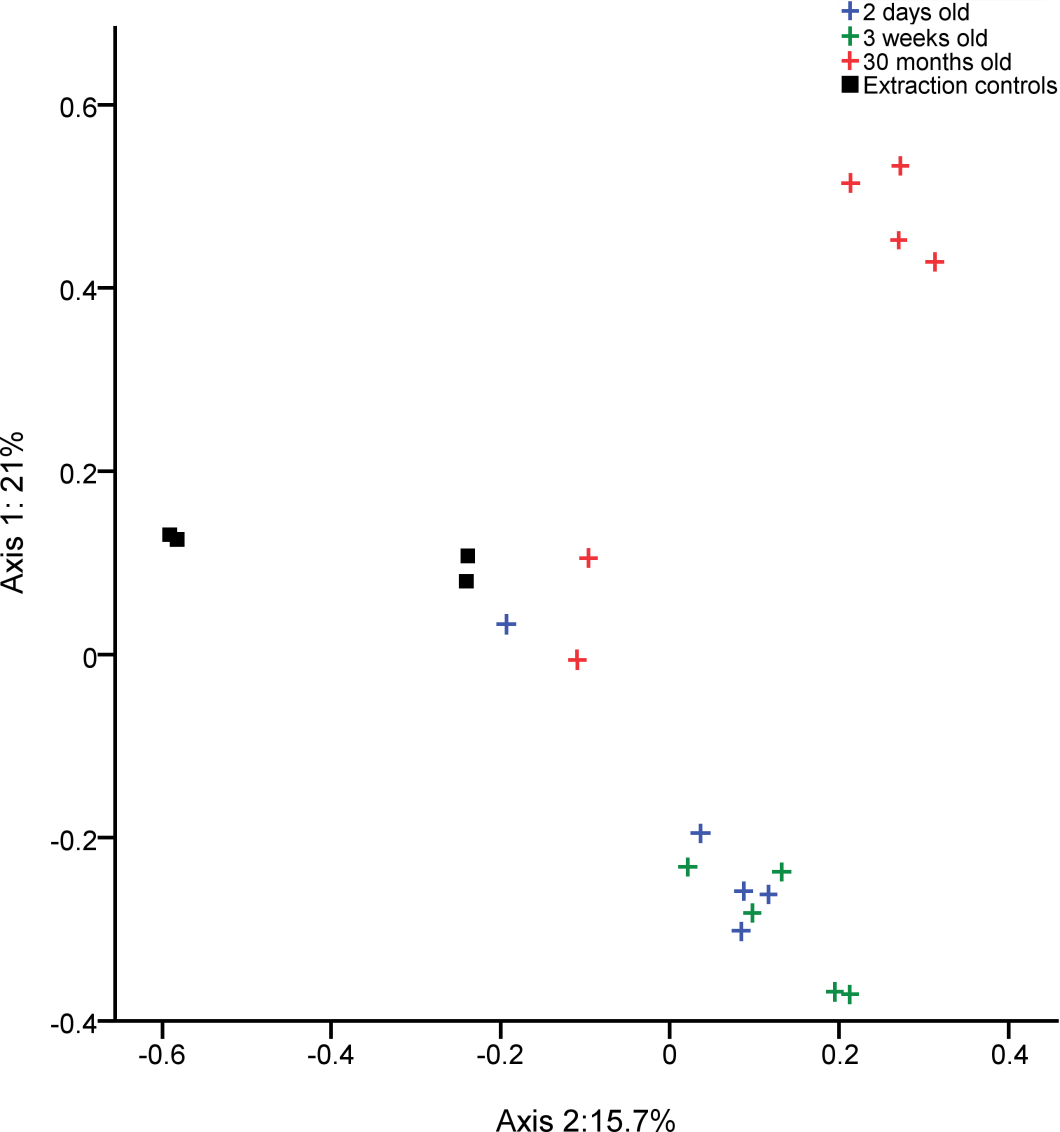
Clustering of chicken nasal swab samples according to age. PCOA graph showing the significantly separate clustering by community composition of the bacterial communities in nasal swabs from chickens of different ages (AMOVA: P<0.001). Each axis label shows the total percentage of variability between samples which is represented by that axis.

The most common bacterial OTUs found on average in nasal swabs were *Staphylococcus* (8.0%), *Lactobacillus* (6.2%), Enterobacteriaceae (6.0%), *Faecalibacterium prausnitzii* (5.0%), *Staphylococcus equorum* (5.0%) and *Lactobacillus reuteri* (4.4%) (Fig 4). Samples clustered by their bacterial community compositions according to age group (AMOVA: P<0.001) (Fig 5). The richness of these bacterial communities was also significantly different between age groups (Kruskal-Wallis Test: P = 0.01), with richness rising with age, but age groups did not differ significantly in diversity. Bacterial OTUs which are indicative of specific age groups can be found in S5 Table.

### Lung microbiota

The most common bacterial OTUs found on average in BAL fluid samples were *Pseudomonas* (20.7%), *Achromobacter* (4.8%), *Lactobacillus* (4.8%), *Turicibacter* (4.7%), *SMB53* (3.6%) and *Lactobacillus* (3.0%). Due to the low biomass of lung microbiota samples, they are sensitive to contamination from bacterial DNA originating from reagents. As such PBS only negative controls were also analysed. While the bacterial community compositions in BAL fluid samples were significantly different from PBS controls (AMOVA: P = 0.024) the most common bacterial OTU on average in PBS controls was identified as *Pseudomonas* (29.7%). This OTU occurred at high abundance in some of our samples and is likely to be due to contamination.

**Fig 6:**
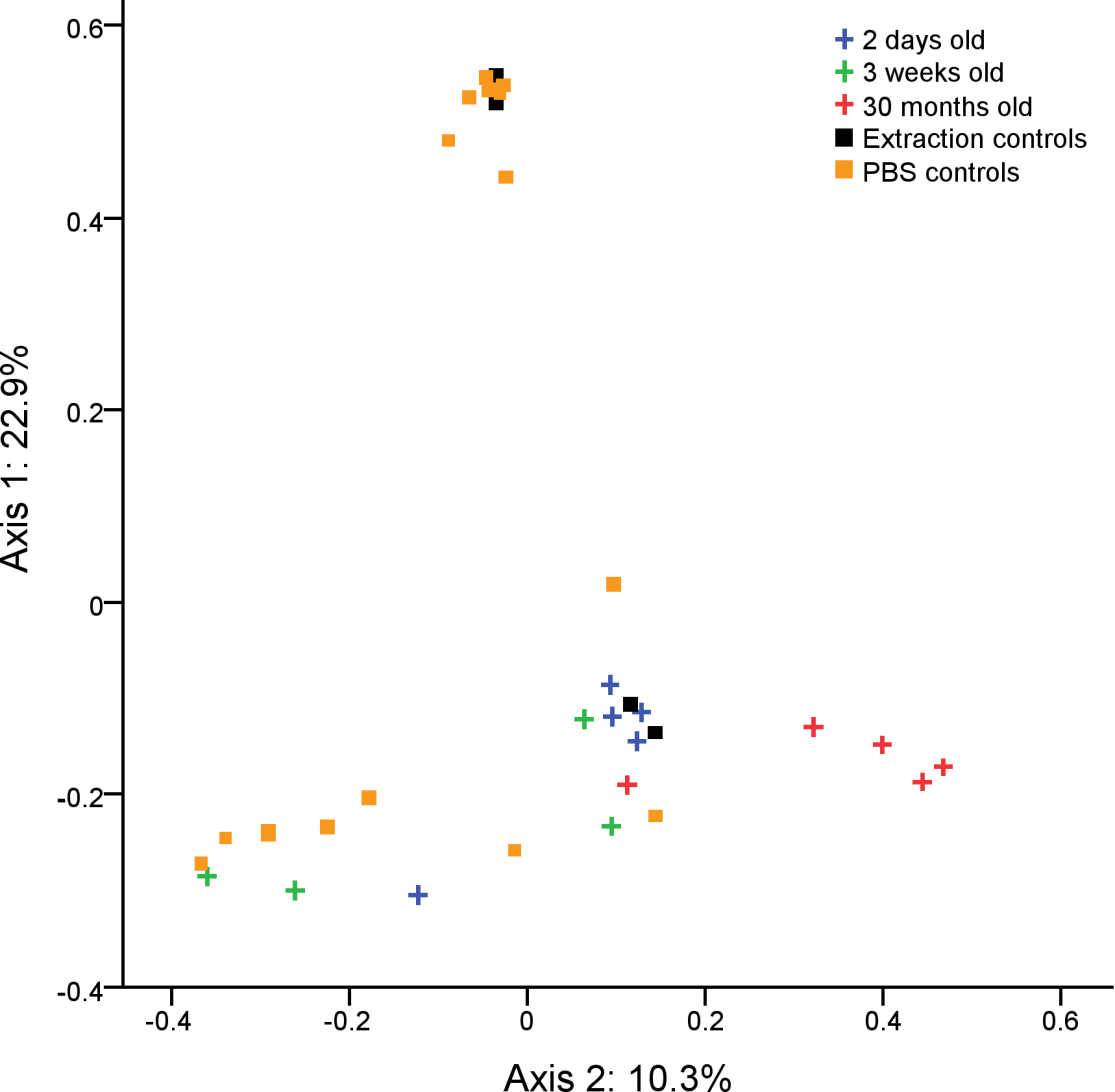
Clustering of chicken BAL fluid samples according to age. PCOA graph showing the significantly separate clustering by community composition of the bacterial communities in BAL fluid from chickens of different ages (AMOVA: P = 0.017). Prior to clustering, OTUs identified as *Pseudomonas* were removed as their presence was likely due to contamination from reagents. Each axis label shows the total percentage of variability between samples which is represented by that axis.

**Fig 7:**
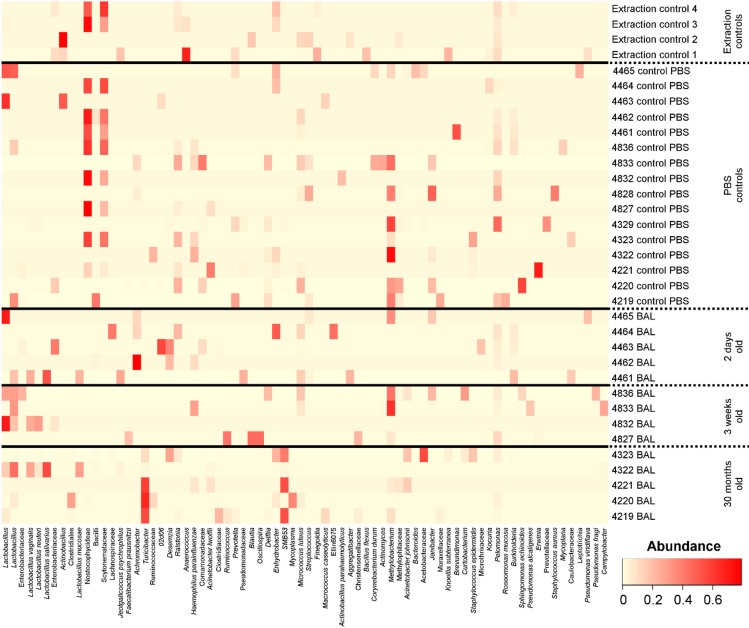
Heatmap of BAL fluid samples grouped by animal age. Bacterial OTUs were included where they had an abundance of ≥7% in at least one sample. OTUs labelled *Pseudomonas* have been removed as their presence was likely due to reagent contamination. DNA extraction kit reagent controls are labelled as Extraction control *n*. PBS controls are labelled as *n* control PBS, where *n* is the chicken sampled immediately after the control sample was taken.

Initially, samples did not cluster significantly by age group according to their bacterial community compositions, richness or diversity. However, when the *Pseudomonas* OTU was removed samples did cluster by age group according to their bacterial community compositions (AMOVA: 0.017) (Fig 6). Several other bacterial OTUs were identified in our reagent controls (at lower abundance) but were not commonly found in our BAL samples (Fig 7) and were therefore not removed prior to statistical analysis. The 30 month age group clustered significantly separately from both the 2 day age group (AMOVA: p = 0.045) and the 3 week age group (AMOVA: p = 0.031); however, the 2 day and 3 week samples did not cluster significantly separately from one another.

After *Pseudomonas* removal, the most common bacterial OTUs found on average in BAL fluid samples were *Lactobacillus* (7.4%), *Turicibacter* (6.6%), *Achromobacter* (5.2%), *Methylobacterium* (4.8%), *SMB53* (4.7%) and *Lactobacillus* (4.3%) (Fig 7). The most common bacterial OTUs found on average in PBS control samples were Nostocophycideae (17.5%), Scytonemataceae (9.6%), Methylobacterium (8.6%), *Pelomonas* (3.9%), *Lactobacillus* (3.8%) and *Erwinia* (2.5%). The following OTUs were found to be significantly indicative (P<0.05) of BAL samples from the 30 month samples: *Corynebacterium* (P = 0.032), *Clostridium perfringens* (P = 0.028), Fusobacteriaceae (P = 0.02), Clostridiales (P = 0.012), *Turicibacter* (P = 0.008), Peptostreptococcaceae (P = 0.004), *SMB53* (P = 0.004) and *Fusobacterium* (P<0.001). No OTUs were found to be significantly indicative of samples from the 2 day or 3 week age groups.

## Discussion

This is the first published study to compare the microbiota at multiple respiratory sites of chickens from different age groups using 16S rRNA gene analysis. We found that there were significant differences in the bacterial communities identified in buccal, nasal and BAL fluid samples. We also observed differences in the bacterial communities at each of these sites, based upon the age of the chickens from which the samples were taken. Significant differences in the richness and diversity of these communities was also observed between age groups.

By far the most common bacteria identified in our buccal swab samples were members of the genus *Lactobacillus*. Lactobacilli are known to be common colonisers of the chicken respiratory tract [13,14,23,24] along with members of the family Enterobacteriaceae which were also found in high abundance in our older birds. This confirms that by using 16S rRNA gene analysis it is possible to identify common respiratory colonisers which have previously been identified using culture based techniques. We also found a high abundance of lactobacilli in the nasal swabs from both the 2 day and 3 week old birds. However, the 30 month old birds showed a far lower abundance of lactobacilli and instead *Jeotgalicoccus*, *Staphylococcus* and *smb53* were the most abundant bacteria.

Previously, lung microbiota samples have been shown to be affected by contaminating bacterial DNA originating from sterile lab reagents and equipment, due to the low bacterial biomass these samples contain [25]. As such it is recommended that reagent controls are processed alongside samples in lung microbiota studies [26]. Previous studies have routinely identified low concentrations of bacterial DNA in “reagent only” controls, often originating from skin-colonising and environmental bacteria [25,27–29]. As expected, our BAL fluid samples were affected by contaminating bacterial DNA originating from the PBS which was used to collect these samples, notably *Pseudomonas* which was found in relatively high abundance in our PBS controls and in some of our BAL samples. The high abundance of *Pseudomonas* in our controls, along with the fact that *Pseudomonas* spp. are common environmental bacteria led us to remove *Pseudomonas* from our BAL samples prior to statistical analysis as we felt confident that the presence of these sequences was due to contamination. Other OTUs found in our reagent controls were not commonly found in our BAL samples and were therefore not removed prior to statistical analysis. After removing *Pseudomonas*, there was a large amount of variation in the bacterial communities isolated from the BAL fluid of different birds, both between age groups and within age groups. Despite this, after *Pseudomonas* was removed from our analysis the oldest age group did cluster significantly separately from the other age groups by its bacterial community structure. Interestingly, while in humans the lung microbiota is often highly similar to the oral microbiota [30,31] this is not the case in our chickens as the types of bacteria found in BAL fluids were significantly different from those found in both buccal and nasal swabs. This may be due to differences between the human and avian respiratory systems or due to the different environmental conditions to which these species are exposed.

While the presence of Staphylococci, Lactobacilli and members of the Enterobacteriaceae corresponds with previous findings from culture based studies, we identified several bacteria which were in ≥5% abundance in at least one of our respiratory samples which had previously not been identified in high abundance in culture based studies. These include several bacteria which have previously been found as members of the chicken gut microbiota, such as *Faecalibacterium*, *Enterococcus cecorum*, *Turicibacter* and *smb53* [4,32–34], and bacteria which have previously been isolated from poultry house air, such as *Jeotgalicoccus* [35]. Several of the bacteria which we isolated were also found in a previous study of chicken BAL fluid using 16S rRNA gene analysis, including *Gallibacterium*, *Avibacterium*, *Acinetobacter* and *Staphylococcus* [2].

The avian respiratory tract is the common site of pathogen entry and disease, including Newcastle disease, infectious bronchitis, and avian influenza. The treatment and prevention of respiratory infections are of utmost importance for the industry, not only because they have a devastating effect on the poultry flocks, but they also render flocks immunosuppressed and susceptible to opportunistic infections such as colibacillosis. Broilers and layer hens are therefore subject to intensive vaccination regimes and the standard route of vaccination is via spray or eye/nose drop. Changes in the composition of the respiratory microbiota in mammals have been shown to be correlated with various respiratory diseases and to vaccination against specific respiratory pathogens [9,10,26].

This study shows that using 16S rRNA gene analysis to study the chicken respiratory microbiota can allow us to detect the presence of bacteria which may be missed in culture based studies. It provides a baseline on which future studies can be built and demonstrates differences between the respiratory microbiota of chickens at different ages.

## Supporting information

S1 Table Management of chickens at the National Avian Research Facility. (DOCX)

S2 Table Chicken vaccination schedule. (DOCX)

S3 Table Bacterial OTUs, sequenced by Miseq, assigned to samples taken from sheep lungs and controls. OTUs were assigned and classified using MOTHUR http://www.mothur.org/wiki/Main_Page. OTUs were subsampled to 32670.
(XLSX)

S4 Table Significantly indicative OTUs for specific age groups in buccal samples. (XLSX)

S5 Table Significantly indicative OTUs for specific age groups in nasal samples. (XLSX)
