# Supplementary material for: Age-related differences in the respiratory microbiota of chickens
Source: PLoS One. 2017 Nov 22;12(11):e0188455. doi: 10.1371/journal.pone.0188455 (PMC5699826; doi:10.1371/journal.pone.0188455)
Supplement: S1 Table — (DOCX) [file pone.0188455.s001.docx]

**S1 Table. Management of chickens at the National Avian Research Facility.**

| Lighting scheme | 14 daylight 10 dark; 20 lux* |
| --- | --- |
| Bedding | Wood shavings; (Stevenson Bros. Bo Ness, Scotland) |
| Feed 0–6 wks Starter crumb | 189 g of CP/kg; 11.48 MJ of ME/kg (BOCM Pauls. Farmgate Range) |
| Feed 6-16 wks Rearer pellet | 160 g of CP/kg; 11.05 MJ of ME/kg (BOCM Pauls. Bulk delivery) |
| Feed 16+ wks Breeder pellet | 150 g of CP/kg; 10.48 MJ of ME/kg (BOCM Pauls. Bulk delivery) |
| Maximum stocking density | As per code of practice for bird weight |
| Temperature | Gradually reduced from 25 to 20°C |
| Humidity | ~45% |

*Lowered to 12-14 lux in agreement with HO inspector to reduce injurious pecking.
