# Supplementary material for: Age-related differences in the respiratory microbiota of chickens
Source: PLoS One. 2017 Nov 22;12(11):e0188455. doi: 10.1371/journal.pone.0188455 (PMC5699826; doi:10.1371/journal.pone.0188455)
Supplement: S2 Table — (DOCX) [file pone.0188455.s002.docx]

**S2 Table. Chicken vaccination schedule.**

| **Disease** | **Vaccine** | **Age** | **Route** |
| --- | --- | --- | --- |
| Marek's Disease | Marek's Disease Vaccine (Rispens CVI 988) | 1 day | Intramuscular |
| Coccidiosis | Hipracox | 1-7 days | Drinking water |
| Newcastle Disease | Poulvac NDW (Clone 30) | 1-3 weeks | Drinking water |
| Infectious Bronchitis | Poulvac IB Primer (H120 and D274) |  |  |
| Infectious Bursal Disease | Bursine (D78/IBD) | 4-6 weeks | Drinking water |
| Chicken Anemia Virus | Nobilis CAV P4 | 6-9 weeks | Drinking water |
| Infectious Laringotracheitis | Poulvac ILT  (Salsbury strain 146) | 6-12 weeks | Eye drops |
| Infectious Bronchitis | Nobilis IB 4/91 |  | Drinking water |
| Newcastle Disease | Poulvac NDW(Clone 30) |  |  |
| Infectious Bursal Disease | Bursine (D78/IBD) |  |  |
| Avian Encephalomielytis | Encefal-vac (Calnek 1143) | 13-14 weeks | Drinking water |
| Avian Pneumovirus | [Nobilis RT + IBmulti + ND +](http://www.merck-animal-health.com/species/poultry/live-vaccines.aspx) EDS | 16+ weeks | Intramuscular |
| Infectious Bronchitis |  |  |  |
| Newcastle Disease |  |  |  |
| Egg Drop Syndrome |  |  |  |
